# Vitamin D Status and Health Indicators in the Malagasy Population: A Pilot Study

**DOI:** 10.3390/healthcare14070887

**Published:** 2026-03-30

**Authors:** Milos Chudy, Petra Macounova, Nikol Gottfriedova, Adela Novotna, Klara Jaresova, Hana Tomaskova, Rastislav Madar, Marek Buzga

**Affiliations:** 1 Department of Epidemiology and Public Health, Faculty of Medicine, University of Ostrava, 703 00 Ostrava, Czech Republic; petra.macounova@osu.cz (P.M.); nikol.gottfriedova@osu.cz (N.G.); adela.novotna@osu.cz (A.N.); klara.jaresova@osu.cz (K.J.); hana.tomaskova@osu.cz (H.T.); rastislav.madar@osu.cz (R.M.); 2 Department of Neurology, University Hospital Ostrava, 708 52 Ostrava, Czech Republic; 3 Department of Physiology and Pathophysiology, Faculty of Medicine, University of Ostrava, 703 00 Ostrava, Czech Republic; marek.buzga@osu.cz; 4 Institute of Laboratory Medicine, University Hospital Ostrava, 708 52 Ostrava, Czech Republic

**Keywords:** cholesterol, glycemia, madagascar, screening, vitamin D

## Abstract

**Background:** Vitamin D plays an important role in overall health. This study aimed to conduct a pilot screening of serum vitamin D levels in a Malagasy cohort and to compare vitamin D status groups with selected health indicators. **Methods:** A cross-sectional observational pilot study was performed in two geographically distinct regions of Madagascar—a coastal area and an inland area. In total, 150 individuals underwent a single health screening, including semi-quantitative assessment of serum 25-hydroxyvitamin D, as well as evaluation of glycemic and cholesterol levels, blood pressure, anthropometric parameters, and a brief personal and lifestyle questionnaire. **Results:** A total of 148 participants (aged 18–88 years) were analyzed. 45.9% of participants had low serum vitamin D levels (<75 nmol/L). Lower vitamin D levels and higher total cholesterol were observed in the coastal group compared to the inland group (*p* < 0.05). No significant differences were found for most other examined health indicators. In multivariable analysis, age was identified as an important determinant of several outcomes. Vitamin D status did not remain an independent predictor; however, a trend toward an independent association with hypercholesterolemia was observed (*p* = 0.07), while the association with hyperglycemia was less pronounced (*p* = 0.11). **Conclusions:** A substantial proportion of participants exhibited low vitamin D levels despite favorable geographic conditions. The results suggest a potential relationship between vitamin D status and lipid metabolism, although this association did not reach statistical significance after adjustment. These findings provide initial insight into vitamin D status and its potential associations in this setting and may inform future research and public health monitoring.

## 1. Introduction

Madagascar is the fourth largest island in the world, known for its unique biodiversity and rich culture, but its inhabitants face a number of health challenges. The country struggles with high rates of malnutrition [1], infectious diseases [2,3] and limited access to healthcare [4]. The ongoing pandemic of COVID-19 [5] has also put some pressure on health. Against the backdrop of these challenges, it is important to examine the various determinants of health that may affect the health and morbidity of the population.

Vitamin D is a key element in maintaining bone health and modulating the immune system. Its sufficiency may have a positive effect on the prevention and prognosis of many chronic diseases [6,7]. Hypovitaminosis of vitamin D is associated with higher insulin resistance, hypercholesterolemia, and metabolic syndrome [6,8]. Low serum vitamin D levels have also been observed more frequently in patients with muscle pain, widespread chronic pain, or arthritis [9]. Poorer sleep quality has also been associated with low vitamin D levels; sleep quality itself is a major factor affecting the health of the body [10,11]. Furthermore, sufficient levels of vitamin D also positively affect fatigue or psychological state of an individual [12,13]. An interesting aspect is the geographical location of Madagascar, most of whose territory is located above the Tropic of Capricorn (23°26′14.440″ south latitude), a strategic position away from endogenous vitamin D synthesis. Countries above, or below, 33° latitude are very limited in time in terms of endogenous vitamin D synthesis in humans. Inhabitants of these northern and southern countries often cannot produce vitamin D3 in their skin for up to 6 months of the year [7]. The main source of vitamin D is endogenous synthesis in the skin, with another secondary source being the dietary intake of vitamin D or supplementation. Fish, egg yolk, and viscera, such as the liver, are very good sources of vitamin D3. Furthermore, some wild mushrooms may contain high amounts of vitamin D2 [6,14].

Despite this natural geographic advantage, the actual serum levels of vitamin D in individuals in Madagascar and their relationship with selected health indicators remain unclear. Therefore, the aim of this pilot study was to analyze serum vitamin D levels in Malagasy participants and to compare them with selected indicators of health and disease.

## 2. Materials and Methods

**Figure 1:**
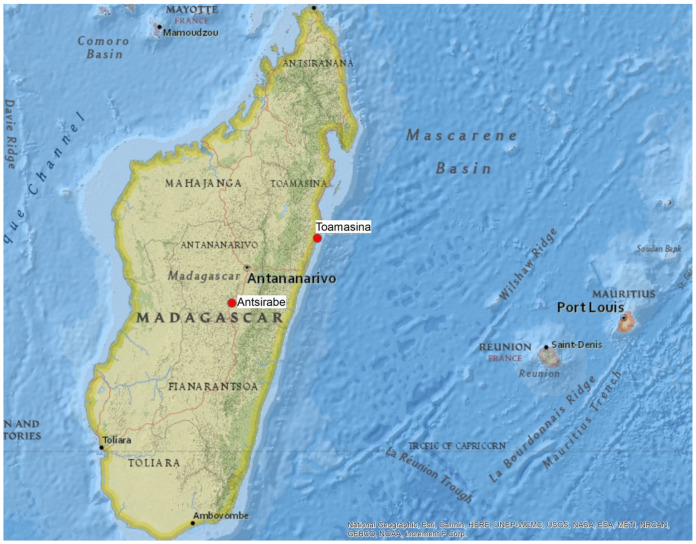
Location of the towns of Antsirabe and Toamasina [National Geographic, Esri].

A cross-sectional observational pilot study was conducted in two geographically distinct regions of Madagascar: a coastal area (Toamasina) and an inland area (Antsirabe) (Figure 1). A total of 150 individuals participated, with equal numbers recruited from each site. Data collection took place during the winter period (June–July).

Participants underwent a single health screening that included measurement of serum vitamin D levels, capillary blood glucose and total cholesterol, blood pressure, height, and weight. The examination was supplemented by a short questionnaire survey.

Participants were recruited through an open call for voluntary participation within local communities. Information about the study was disseminated with the assistance of community workers and local communication channels. Participation was entirely voluntary, and the health examination was provided free of charge. Individuals from the local population who expressed interest in undergoing a health check were eligible to participate. This approach resulted in a non-random sample of individuals who voluntarily participated in the screening.

**Figure 2:**
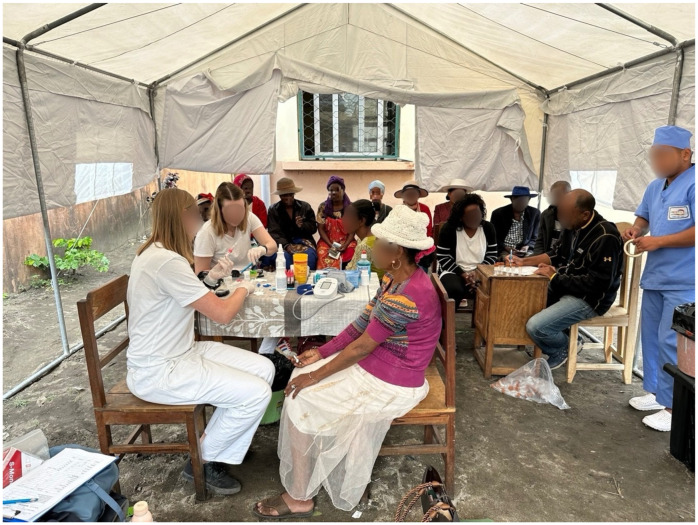
Course of examination at the local clinic [from the author’s archive].

The examinations and data collection were carried out by trained healthcare personnel in local clinical facilities (Figure 2). Participants were informed in advance about the study procedures and were instructed to attend the examination in the morning in a fasting state and to avoid heavy fatty meals the day before testing.

No specific inclusion criteria were applied beyond voluntary participation. Participants were excluded from further analysis if they met any of the following criteria: (i) age <18 years; or (ii) incomplete or inconclusive test results. Based on these criteria, two participants were excluded.

The sample size was determined by the feasibility of data collection during the field study, including available resources and the number of diagnostic tests, and reflects the exploratory nature of this pilot study.

### 2.1. Data Collection

All participants were measured for height (cm) and weight (kg). Based on these measurements, body mass index (BMI) was calculated for each participant. A Silvercrest electronic digital scale (Lidl Stiftung & Co. KG, Neckarsulm, Germany) was used to measure weight. Blood pressure was measured at two time intervals using an automatic upper arm blood pressure monitor M2 Basic: HEM-7121J-E (OMRON Healthcare Co., Muko, Japan) with an accuracy of ±3 mmHg of the displayed value. Hypertension was defined as systolic blood pressure ≥140 mmHg and/or diastolic blood pressure ≥90 mmHg [15].

### 2.2. Biochemical Testing

Serum levels of vitamin D, glycemia and total cholesterol were analyzed. The Vitamin D Rapid Test Cassette (H&W Biotech Oy, Espoo, Finland) was used to determine serum vitamin D levels. It is a rapid chromatographic immunoassay for the semi-quantitative detection of 25-hydroxyvitamin D (25-OH D) in human fingerstick whole blood. Based on the strength of the test strip staining, the test was evaluated as vitamin D deficient (0–24.9 nmol/L), insufficient (25–74.9 nmol/L) and sufficient (75–250 nmol/L). The overall accuracy of the test was 94.4%. Vitamin D status was categorized as sufficient (≥75 nmol/L) and insufficient (<75 nmol/L), in accordance with Endocrine Society clinical practice guidelines [16,17].

The Wellion LUNA trio (Med Trust, Marz, Austria) was used to determine glycemia and total cholesterol. The principle of the measurement method was electrochemical biosensor technology. The instrument was calibrated to plasma reference instruments that were linked to subsequent reference standard materials and methods: glucose standard (NIST SRM 917) and glucose oxidase method; total cholesterol standard (NIST SRM 911) and the Abell/Kendall method. The measurement range for glycemia was 1.1–33.3 mmol/L, with values ≥5.6 mmol/L considered elevated (hyperglycemia) [18]. The range of total cholesterol measurements was 2.6 to 10.3 mmol/L, with total cholesterol values below 2.6 designated as “low.” Respondents with such low cholesterol levels (n = 13) were excluded from some analyses involving metric data. Total cholesterol values ≥5 mmol/L were considered elevated (hypercholesterolemia) [19].

The use of rapid point-of-care testing in this study was motivated by the field-based design and the limited availability of laboratory infrastructure in the study regions. Although these methods may have lower analytical precision compared with standard laboratory-based assays, they represent a practical and feasible solution for data collection in field conditions and are considered sufficient for the screening of selected health indicators [20,21].

### 2.3. The Short-Version Screening Questionnaire

The screening questionnaire was administered as part of the medical history-taking process and focused on symptoms and lifestyle factors potentially associated with low vitamin D levels. The questionnaire was developed by the research team specifically for the purposes of this field-based pilot study.

In addition to age, alcohol consumption, and tobacco use, the questionnaire included questions on the presence of joint and muscle pain, and the presence and frequency (0 = not at all; 1 = occasionally (1–2 times per week); 2 = often (3–6 times per week); 3 = daily) of fatigue, insomnia, sleep problems, nervousness, and mood swings. It also assessed the consumption of selected foods (fish, dairy products, eggs, and fried foods).

The questionnaire was administered with the assistance of a local interpreter who was present throughout the data collection. The interpreter translated the questions into the local language/dialect (Malagasy), respondents answered orally, and the investigating healthcare worker recorded the responses.

### 2.4. Statistical Analyses

Basic descriptive statistics was used to process the data. The Chi-square test or Fisher’s exact test was used for the statistical analysis of the data. Student’s *t* test and the nonparametric Mann–Whitney test were used for comparison of metric variables. The normality of the data was checked using the Shapiro–Wilk test. In addition, multivariable logistic regression analyses were performed to evaluate the independent associations between selected variables. The models were adjusted for age and BMI, and the results are presented as odds ratios (OR) with 95% confidence intervals. Statistical analysis was performed using Stata v. 13.1. (StataCorp LLC., College Station, TX, USA) The tests were evaluated at a significance level of 5%. Tables and graphs were created in MS Office Excel (version 2405) (Microsoft Corporation, Redmond, WA, USA).

## 3. Results

In collaboration with local clinics, a total of 150 individuals were screened, 75 (50%) from the inland area (Antsirabe) and 75 (50%) from the coastal area (Toamasina). Based on predefined exclusion criteria, 2 individuals were excluded prior to statistical analyses. The final study group comprised 148 participants with a mean age of 45.4 ± 17.8 years (min 18; max 88). A statistically significant difference in age between the inland and coastal groups was observed (*p* < 0.001), with a higher proportion of younger individuals in the coastal area (39.8 ± 16.7 years) compared to the inland area (51.0 ± 17.2 years). Females (107; 72.3%) were more frequently represented, accounting for 81.1% of the coastal group and 63.5% of the inland group. Baseline characteristics are presented in Table 1.

Sex-related differences were observed in several characteristics across both groups. The mean height was 156.4 ± 7.9 cm, with no significant differences between sexes; however, individuals in the inland group were significantly taller (*p* = 0.019). The mean weight was 58.5 ± 12.7 kg, with no significant differences between the groups. The mean BMI in the cohort was 23.9 ± 4.8, with no differences between regions or between sexes.

Vitamin D deficiency was significantly more frequent in the coastal group compared to the inland group (*p* < 0.001).

A higher prevalence of hypertension was observed in the inland group (*p* = 0.013). More than half of the cohort had elevated blood pressure (i.e., systolic pressure ≥ 140 mmHg, diastolic pressure ≥ 90 mmHg). Among the 75 cases of hypertension, 15 were isolated systolic and 18 isolated diastolic.

Alcohol consumption and smoking were infrequent. Only 3 individuals (2%) reported smoking, and 20 (13.5%) reported alcohol consumption, of whom 19 reported a frequency of less than once per week; therefore, these factors were not included in further analyses. No significant differences in reported dietary habits were observed between regions, sexes, or vitamin D status groups.

### 3.1. The Short-Version Screening Questionnaire

As part of the screening questionnaire, individuals were asked about the presence and/or frequency of selected risk factors associated with low vitamin D levels, and responses were compared between coastal and inland groups.

More than half (56.1%) reported joint and muscle pain, with a non-significantly higher prevalence in the coastal group and among females. Fatigue was reported by 115 individuals (77.7%), with no significant difference between regions. However, a difference was observed in frequency (*p* < 0.001), with daily fatigue reported more frequently in the inland group (16; 21.6%) compared to the coastal group (2; 2.7%). Females were significantly more likely to report fatigue than males (82.2% vs. 65.9%, *p* = 0.032), and both daily and frequent fatigue were more common among women. Sleep disturbances were reported by 62 individuals (41.9%), with a significantly higher prevalence in the inland group (*p* = 0.019), although no differences were observed in frequency (*p* = 0.122). No differences were found between sexes in either prevalence or frequency of sleep disturbances (*p* = 0.237 and *p* = 0.651, respectively). Mood fluctuations, irritability, or nervousness were reported by 65 individuals (43.9%). No significant differences were observed between regions in either prevalence or frequency (*p* = 0.408 and *p* = 0.156, respectively), although these symptoms were more frequently reported by females (*p* = 0.026).

### 3.2. Vitamin D Levels

**Figure 3:**
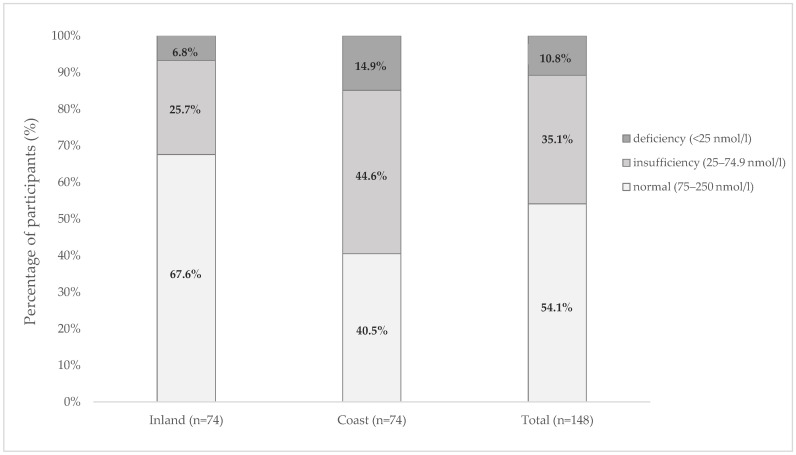
Number of participants in relation to vitamin D levels.

Adequate serum vitamin D levels (≥75 nmol/L) were observed in 80 (54.1%) individuals. A further 52 (35.1%) had insufficient levels (25–75 nmol/L), and 16 (10.8%) had deficient levels (<25 nmol/L) (Figure 3).

Significantly higher serum vitamin D levels were observed in the inland group (*p* < 0.001, Table 1, Figure 3) and among individuals with physiological total cholesterol levels (*p* = 0.034). No significant differences in serum vitamin D levels were observed between sexes or across other examined factors (Table 2).

No additional significant associations with serum vitamin D levels were observed after stratification by sex and region, with the exception of hypercholesterolemia, which was significantly more common in women with low vitamin D levels (Table 3). Pain was more frequently reported among individuals with deficient vitamin D levels compared to those with insufficient or sufficient levels.

Individuals with total cholesterol ≥ 5 mmol/L were significantly more likely to have low vitamin D levels (*p* = 0.034) (Table 2). The mean total cholesterol level was 4.26 ± 1.02 mmol/L (n = 135), while in 13 individuals the values were below the detectable range (<2.6 mmol/L). Overall, 32 individuals (21.6%) had total cholesterol ≥ 5 mmol/L (Table 2). Higher total cholesterol levels were observed in the coastal group compared to the inland group (*p* < 0.001), with 35.1% vs. 8.1% of individuals exceeding 5 mmol/L, respectively. Women more frequently had total cholesterol ≥ 5 mmol/L than men (27.1% vs. 7.3%, *p* = 0.008).

The mean glycemic level was 6.63 ± 3.07 mmol/L. No statistically significant differences were observed between regions; however, glycemic levels differed between sexes (*p* = 0.002). No significant associations were found between glycemic levels and serum vitamin D levels in either the inland (*p* = 0.226) or coastal group (*p* = 0.522). Mean glycemic levels were 7.09 ± 2.23 mmol/L in men and 6.46 ± 3.32 mmol/L in women. Two individuals (1.4%) had values within the hypoglycemic range, while 52 (35.1%) had normal values (3.9–5.5 mmol/L). A total of 64 (43.2%) individuals had values corresponding to prediabetes (5.6–6.9 mmol/L), and 30 (20.3%) had values ≥ 7 mmol/L, consistent with diabetes mellitus.

The mean systolic blood pressure was 137 ± 27 mmHg in individuals with sufficient vitamin D levels and 132 ± 26 mmHg in those with levels < 75 nmol/L. The mean BMI was 23.9 ± 4.3 in individuals with sufficient vitamin D levels and 23.9 ± 5.3 in those with low levels. No significant associations were observed between vitamin D status and the presence of hypertension or BMI.

### 3.3. Multivariable Analysis

To account for potential confounding factors, multivariable logistic regression analyses were performed, adjusting for age and BMI. The detailed results are presented in Table 4.

In the model assessing hypercholesterolemia, no statistically significant independent association was observed between vitamin D status and hypercholesterolemia after adjustment (*p* = 0.066). Neither age nor BMI were significant predictors in this model.

In the model assessing hyperglycemia, age was identified as a significant independent predictor (*p* = 0.002), while vitamin D status was not significantly associated with hyperglycemia (*p* = 0.111). Cholesterol levels were also independently associated with hyperglycemia (*p* = 0.031).

In the model assessing hypertension, age (*p* < 0.001) and BMI (*p* = 0.027) were significant predictors, whereas vitamin D status was not independently associated with hypertension (*p* = 0.402).

## 4. Discussion

A total of 45.9% of participants had low serum vitamin D levels, although Madagascar’s geographical location can be considered advantageous in terms of sunlight availability. According to Lips et al., vitamin D status varies between continents and countries [22]. In Australia and Latin America, vitamin D status is generally adequate [22,23], but in the Middle East and some Asian countries, it is very low [22,23]. According to Mogire et al. who examined the prevalence of low vitamin D levels in Africa, the overall prevalence was 18.46% at a 25-OH D concentration threshold below 30 nmol/L; 34.22% at a threshold below 50 nmol/L; and 59.54% at a threshold below 75 nmol/L [24]. The mean concentration of 25-OH D was 67.78 nmol/L, with lower concentrations in populations living in North Africa as well as South Africa compared to sub-Saharan Africa [24], which is consistent with our findings. The authors also found that vitamin D levels were lower in urban areas compared to rural areas, and lower in women compared to men [24]. In contrast, our study included only participants from rural areas rather than urban settings; however, within our sample, a significant difference in vitamin D deficiency was observed between the studied regions, with a higher prevalence among coastal participants. No similar differences were observed between men and women. In addition to environmental factors such as sunlight exposure, dietary intake may also contribute to vitamin D status. In our study, selected dietary habits, including the consumption of fish, dairy products, and eggs, were assessed as part of the screening questionnaire; however, only frequency of consumption was recorded without quantitative assessment of portion size or actual nutrient intake. No significant differences in reported dietary habits were observed between the studied groups. Therefore, although diet may play a role in vitamin D status, its impact could not be evaluated in detail in our study. The observed differences in vitamin D levels in the coastal region may be related to environmental factors such as weather conditions, as this area is characterized by frequent rainfall and high cloud cover. In addition to the inland–coastal distinction, the regions differ in several meteorological characteristics based on long-term climatological data. In the inland city of Antsirabe, the average minimum temperature in June is 6.7 °C and the maximum 21.2 °C. In July, these values are similar with 5.9 °C and 21 °C. In the coastal city of Toamasina, the minimum average temperatures in June are 18 °C and the maximum 26.1 °C. In July, these values are 17 °C and 25 °C respectively. The number of rainfall days also varies, with the average number of rainfall days in June and July in Toamasina being 21 and 24 days, respectively. In Antsirabe, the average number of days of rain in both months is only 4 [25].

Trends in vitamin D levels can be predictors and indicators of general health status [22]. One of the findings of our study was an association between low serum vitamin D levels and hypercholesterolemia. To further explore these associations, multivariable logistic regression analyses adjusted for age and BMI were performed. After adjustment, the association between vitamin D status and hypercholesterolemia did not reach statistical significance; however, a consistent trend was observed, suggesting a potential relationship between lower vitamin D levels and an increased likelihood of hypercholesterolemia. Qin et al. reported that vitamin D supplementation was associated with improved serum lipid levels in patients with hypercholesterolemia, including lower triglyceride and total cholesterol levels [8]. A systematic review and meta-analysis by Dibaba et al. also reported beneficial effects of vitamin D supplementation on total cholesterol, LDL cholesterol, and triglycerides, while no effect on HDL cholesterol levels was observed [26].

Several biological mechanisms have been proposed to explain the association between vitamin D status and lipid profile. Vitamin D acts through the vitamin D receptor and is involved in the regulation of gene expression and inflammatory pathways, both of which are linked to metabolic processes. In addition, previous studies have reported associations between low vitamin D levels and a more atherogenic lipid profile, including higher levels of total cholesterol, LDL cholesterol, and triglycerides. However, the exact mechanisms underlying this relationship remain incompletely understood [27]. At the same time, vitamin D should be considered as only one of many factors that may influence cholesterol levels, alongside diet, physical activity, adiposity, and other metabolic and environmental determinants.

In our cohort, no significant differences in the incidence of hyperglycemia were observed with respect to vitamin D status. However, multivariable analysis adjusted for age and BMI suggested a possible association between vitamin D status and hyperglycemia, although the relationship did not reach statistical significance. Age was identified as a significant independent predictor, indicating that the observed patterns may be partly driven by age-related factors. In addition, hypercholesterolemia was identified as an independent factor associated with hyperglycemia in the multivariable model, which is consistent with the known clustering of metabolic risk factors. The authors Alaei-Shahmiri et al. reported that individuals with hyperglycemia in an Iranian male population had significantly lower vitamin D levels than those with normal glycemic levels [28]. An association with low vitamin D levels and hyperglycemia has also been described in populations in northern latitudes [29].

According to a study by Weishaar et al., skin color and body weight also influence vitamin D levels [30]. The mean weight in our cohort was 58.5 ± 12.7 kg. No differences were observed between the study groups in terms of weight or BMI values. In the case of BMI, a slight, nonsignificant trend of increasing BMI with decreasing vitamin D levels was observed in the inland group.

One of the most common causes of death in Madagascar is stroke [31,32], making Madagascar one of the countries with the highest stroke mortality rates worldwide, along with Mongolia [33]. Hypertension is an important risk factor for stroke or myocardial infarction and was frequently observed. A meta-analysis by Qi et al. showed that vitamin D supplementation may modestly contribute to the reduction in systolic blood pressure [34], although vitamin D itself is not an antihypertensive agent [34]. In our study, there was no association between serum vitamin D levels and blood pressure, although a slight non-significant trend of increased systolic blood pressure with decreasing serum vitamin D levels was observed among coastal participants, who also showed a higher proportion of hypertensive individuals compared to inland participants. Consistent with these findings, multivariable analysis adjusted for age and BMI did not indicate an independent association between vitamin D status and hypertension. In contrast, age and BMI were identified as significant predictors, suggesting that blood pressure levels in this cohort are more strongly influenced by established cardiovascular risk factors than by vitamin D status.

Inland participants were more likely to report insomnia compared to coastal participants and were also more likely to experience fatigue. Oliveira et al. reported that vitamin D supplementation was associated with improved sleep quality and reduced pain, particularly in individuals with vitamin D deficiency [35]. They also reported an inverse correlation between vitamin D levels and pain [35]. Although differences were observed between our study groups, their association with vitamin D levels was not statistically significant.

The results of this pilot screening indicate that more than 32% of inland participants and nearly 60% of coastal participants in this sample had low serum vitamin D levels. Despite the favorable geographic location of Madagascar, a substantial proportion of participants in this sample exhibited vitamin D deficiency. An association between low vitamin D levels and higher total cholesterol was observed. Further research is needed to better elucidate the relationship between vitamin D status and cholesterol levels, as well as other factors for which non-significant trends were identified in this sample. Given the exploratory nature of this study, these findings should be interpreted as preliminary and hypothesis-generating.

This study has several limitations that should be considered when interpreting the findings. First, this was a pilot, cross-sectional study with an exploratory design and a relatively small sample size, which limits statistical power and the ability to detect some potentially meaningful associations. Participants were recruited through voluntary community-based screening in only two selected localities; therefore, the sample should be considered a convenience sample and may not be fully representative of the broader Malagasy population. The findings should thus not be directly generalized, and the cross-sectional design precludes any causal interpretation.

Another limitation is the use of rapid point-of-care tests, including a semi-quantitative assay for vitamin D and quantitative measurements for glycemia and total cholesterol, which may be associated with greater measurement uncertainty compared with standard laboratory-based methods. However, in this study, these tests were selected due to logistical and economic constraints in a low-resource field setting, where access to advanced laboratory methods is limited.

The interpretation of vitamin D status is further limited by the lack of detailed information on key modifying factors such as sun exposure, clothing habits, occupation, and time spent outdoors. Dietary intake was not the primary focus of this study, and the actual vitamin D intake of participants was not quantified.

Finally, part of the data was based on self-reported questionnaire responses, which may be subject to recall and reporting bias. In addition, the questionnaire was developed by the authors for the purposes of this study and was not formally validated. The translation of the questionnaire was performed in real time by a local interpreter without the use of a forward–backward translation procedure, which may have introduced information bias.

Taken together, these limitations indicate that the findings should be considered preliminary and hypothesis-generating rather than definitive. Nevertheless, the study provides useful initial insight into vitamin D status in a low-resource setting and may support future research and public health considerations in Madagascar.

## 5. Conclusions

In this pilot sample, 45.9% of participants had low serum vitamin D levels. Lower vitamin D levels were observed among coastal participants compared to those from inland areas. An association between low vitamin D levels and higher total cholesterol was observed in univariate analyses; however, this relationship did not remain statistically significant after adjustment for age and BMI, although a trend persisted. No significant differences were observed for most other examined health indicators; however, a non-significant trend was noted, with individuals with low vitamin D levels more frequently reporting muscle and joint pain. Inland participants were more likely to report sleep difficulties.

Given the exploratory nature of the study, these findings should be interpreted with caution and cannot be directly generalized to the broader Malagasy population. Nevertheless, the results provide initial insight into vitamin D status and related health indicators in this setting and may serve as a basis for future research and public health monitoring.

## Figures and Tables

**Table 1:** Characteristics of participants.

Group		Coastal Group	Inland Group	Total	*p*-Values
Number of participants (%)		74 (100)	74 (100)	148 (100)	
Number of women (%)		60 (81.1)	47 (63.5)	107 (72.3)	0.017 ^b^
Mean age ± SD		39.8 ± 16.7	51.0 ± 17.2	45.4 ± 17.7	<0.001 ^a^
Height (mean value) ± SD		155.3 ± 6.68	157.4 ± 8.8	156.4 ± 7.9	0.019 ^c^
Weight (mean value) ± SD		57.4 ± 12.1	59.5 ± 13.4	58.5 ± 12.7	0.888 ^a^
BMI (n; %)	<18.5	7 (9.5)	9 (12.2)	16 (10.8)	0.596 ^b^
	18.5–24.9	35 (47.3)	31 (41.9)	66 (44.6)	0.508 ^b^
	25–29.9	20 (27.0)	27 (36.5)	47 (31.8)	0.216 ^b^
	≥30	8 (10.8)	7 (9.5)	15 (10.1)	0.785 ^b^
Vit D low level (<75 nmol/L) (n; %)		44 (59.5)	24 (32.4)	68 (45.9)	<0.001 ^b^

n = number of participants; SD = standard deviation; BMI = body mass index. (^a^ Nonparametric Mann–Whitney test; ^b^ Chi-square test; ^c^ Student’s *t*-test; significance level 5%).

**Table 2:** Number of participants in individual study groups with respect to vitamin D levels.

All Participants (n = 148)			
Vitamin D levels	75–250 nmol/L	<75 nmol/L	*p*-Value ^a^
	n (%)	n (%)	
Number of participants (%)	80 (100)	68 (100)	-
Inland	50 (62.5)	24 (35.3)	<0.001
Coast	30 (37.5)	44 (64.7)	
Men	21 (26.3)	20 (29.4)	0.668
Women	59 (73.8)	48 (70.6)	
Hyperglycaemia	52 (65.0)	48 (70.6)	0.469
Hypercholesterolemia	12 (15.0)	20 (29.4)	0.034
BMI ≥ 25	35 (43.8)	28 (41.2)	0.752
Hypertension	42 (52.5)	33 (48.5)	0.630
Joint pain	44 (55.0)	39 (57.4)	0.774
Fatigue	61 (76.3)	54 (79.4)	0.645
Sleep problems	34 (42.5)	28 (41.2)	0.871
Nervousness/anxiety	35 (43.8)	30 (44.1)	0.964

n = number of participants. (^a^ Chi-square test; significance level 5%).

**Table 3:** Characteristics of the participants in the individual study groups with respect to vitamin D levels (75–250 nmol/L = normal levels; <75 nmol/L = low levels).

Groups	Men (n = 41)			Women (n = 107)			Coastal (n = 74)			Inland (n = 74)		
Vitamin D Levels	75–250 nmol/L	<75 nmol/L	*p*-Value	75–250 nmol/L	<75 nmol/L	*p*-Value	75–250 nmol/L	<75 nmol/L	*p*-Value	75–250 nmol/L	<75 nmol/L	*p*-Value
Number of participants (%)	21 (100)	20 (100)		59 (100)	48 (100)		30 (100)	44 (100)		50 (100)	24 (100)	
Mean age ± SD	56.1 ± 17.0	46.6 ± 19.6	0.161 ^a^	45.0 ± 17.8	40.7 ± 15.6	0.219 ^a^	41.6 ± 18.1	38.6 ± 15.7	0.610 ^a^	51.8 ± 17.3	49.5 ± 17.2	0.569 ^a^
Hyperglycaemia	15 (71.4)	17 (85.0)	0.453 ^c^	37 (62.7)	31 (64.6)	0.841 ^b^	18 (60.0)	28 (63.6)	0.751 ^b^	34 (68.0)	20 (83.3)	0.263 ^c^
Hypercholesterolemia	2 (9.5)	1 (5.0)	1.000 ^c^	10 (16.9)	19 (39.6)	0.008 ^b^	9 (30.0)	17 (38.6)	0.444 ^b^	3 (6.0)	3 (12.5)	0.382 ^c^
Hypertension	15 (71.4)	12 (60.0)	0.440 ^b^	27 (45.8)	21 (43.8)	0.835 ^b^	12 (40.0)	18 (40.9)	0.937 ^b^	30 (60.0)	15 (62.5)	0.836 ^b^
Joint pain	11 (52.4)	11 (55.0)	0.866 ^b^	33 (55.9)	28 (58.3)	0.802 ^b^	17 (56.7)	29 (65.9)	0.420 ^b^	27 (54.0)	10 (41.7)	0.320 ^b^
Fatigue	12 (57.1)	15 (75.0)	0.228 ^b^	49 (83.1)	39 (81.3)	0.808 ^b^	26 (86.7)	34 (77.3)	0.376 ^c^	35 (70.0)	20 (83.3)	0.266 ^c^
Sleep problems	5 (23.8)	9 (45.0)	0.152 ^b^	29 (49.2)	19 (39.6)	0.322 ^b^	10 (33.3)	14 (31.8)	0.891 ^b^	24 (48.0)	14 (58.3)	0.405 ^b^
Nervousness/anxiety	6 (28.6)	6 (30.0)	0.919 ^b^	29 (49.2)	24 (50.0)	0.930 ^b^	12 (40.0)	18 (40.9)	0.937 ^b^	23 (46.0)	12 (50.0)	0.746 ^b^

SD = standard deviation. (^a^ Nonparametric Mann–Whitney test; ^b^ Chi-square test; ^c^ Fisher test; significance level 5%).

**Table 4:** Multivariable logistic regression models for hypercholesterolemia, hyperglycemia, and hypertension.

**Model A—Hypercholesterolemia**				
	**OR**	**SE**	**95% CI**	***p*** **-value**
Age	0.979	0.012	0.955 to 1.004	0.094
BMI	1.002	0.044	0.921 to 1.092	0.956
Vitamin D status	0.464	0.194	0.205 to 1.052	0.066
**Model B—Hyperglycemia**				
	**OR**	**SE**	**95% CI**	***p*** **-value**
Age	1.036	0.012	1.013 to 1.06	0.002
BMI	1.014	0.041	0.936 to 1.098	0.729
Hypercholesterolemia	0.719	0.110	0.533 to 0.971	0.031
Vitamin D status	0.534	0.211	0.246 to 1.156	0.111
**Model C—Hypertension**				
	**OR**	**SE**	**95% CI**	***p*** **-value**
Age	1.089	0.016	1.059 to 1.121	<0.001
BMI	1.108	0.051	1.012 to 1.213	0.027
Hypercholesterolemia	0.919	0.145	0.673 to 1.254	0.595
Vitamin D status	0.693	0.302	0.295 to 1.630	0.402

Age and body mass index (BMI) were included as continuous variables, while hypercholesterolemia, hyperglycemia, and hypertension were analyzed as binary outcomes (yes/no). Vitamin D status was categorized as sufficient (≥75 nmol/L) and insufficient (<75 nmol/L). OR = odds ratio; SE = standard error; 95% CI = 95% confidence interval.

## Data Availability

The dataset used and analyzed in this study is available from the corresponding author. The data are not publicly available due to privacy concerns related to the nature of the collected health data.

## Abbreviations

The following abbreviations are used in this manuscript:

BMI	body mass index
HDL	high-density lipoprotein
LDL	low-density lipoprotein
SD	standard deviation
25-OH D	25-hydroxyvitamin D
